# Characterization of early pathogenesis in the SOD1^G93A^ mouse model of ALS: part II, results and discussion

**DOI:** 10.1002/brb3.142

**Published:** 2013-06-11

**Authors:** Sharon Vinsant, Carol Mansfield, Ramon Jimenez-Moreno, Victoria Del Gaizo Moore, Masaaki Yoshikawa, Thomas G Hampton, David Prevette, James Caress, Ronald W Oppenheim, Carol Milligan

**Affiliations:** 1 Department of Neurobiology and Anatomy, The Neuroscience Program and The ALS Center, Wake Forest University School of MedicineWinston-Salem, North Carolina; 2 Department of Chemistry, Elon UniversityElon, North Carolina; 3 Mouse SpecificsBoston, Massachusetts; 4 Department of Neurology and the ALS Center, Wake Forest University School of MedicineWinston-Salem, North Carolina

**Keywords:** Axons, cytoplasmic vacuoles, glia, mega-mitochondria, mitochondria, motoneurons, motor function, NMJs

## Abstract

Pathological events are well characterized in amyotrophic lateral sclerosis (ALS) mouse models, but review of the literature fails to identify a specific initiating event that precipitates disease pathology. There is now growing consensus in the field that axon and synapses are first cellular sites of degeneration, but controversy exists over whether axon and synapse loss is initiated autonomously at those sites or by pathology in the cell body, in nonneuronal cells or even in nonmotoneurons (MNs). Previous studies have identified pathological events in the mutant superoxide dismutase 1 (SOD1) models involving spinal cord, peripheral axons, neuromuscular junctions (NMJs), or muscle; however, few studies have systematically examined pathogenesis at multiple sites in the same study. We have performed ultrastructural examination of both central and peripheral components of the neuromuscular system in the SOD1^G93A^ mouse model of ALS. Twenty percent of MNs undergo degeneration by P60, but NMJ innervation in fast fatigable muscles is reduced by 40% by P30. Gait alterations and muscle weakness were also found at P30. There was no change in axonal transport prior to initial NMJ denervation. Mitochondrial morphological changes are observed at P7 and become more prominent with disease progression. At P30 there was a significant decrease in excitatory axo-dendritic and axo-somatic synapses with an increase in C-type axo-somatic synapses. Our study examined early pathology in both peripheral and central neuromuscular system. The muscle denervation is associated with functional motor deficits and begins during the first postnatal month in SOD1^G93A^ mice. Physiological dysfunction and pathology in the mitochondria of synapses and MN soma and dendrites occur, and disease onset in these animals begins more than 2 months earlier than originally thought. This information may be valuable for designing preclinical trials that are more likely to impact disease onset and progression.

## Introduction

Neurodegenerative diseases are unique in the distinct subtype of neuronal populations that selectively undergo cell death. Recent evidence derived from the study of animal models of various neuropathological conditions has revealed that damage to axons and synapses often long precedes the activation of death pathways and the onset of clinical (i.e., functional) pathology (Coleman and Perry 2002; Raff et al. 2002; Medana and Esiri 2003; Palop et al. 2006; Gould and Oppenheim 2007; Saxena and Caroni 2007). Furthermore, in at least some neurodegenerative disease models with early onset of axon/synapse loss, including amyotrophic lateral sclerosis (ALS), protecting cell bodies from death fails to substantially alter disease progression or life span (Sagot et al. 1995; Kostic et al. 1997; Ferri et al. 2003; Chiesa et al. 2005; Libby et al. 2005; Gould et al. 2006; Suzuki et al. 2007). In the case of mouse models of ALS, muscle denervation appears to occur months before motoneuron (MN) death (Frey et al. 2000; Fischer et al. 2004; Gould et al. 2006; Pun et al. 2006). Although there is a paucity of studies of this issue in humans, it appears that denervation also corresponds to early symptom onset in ALS patients (Tsujihata et al. 1984; Siklós et al. 1996; Aggarwal and Nicholson 2002; Fischer et al. 2004; Blijham et al. 2007). Together these results prompted us to further evaluate when and where pathology begins and how it correlates with initial muscle denervation.

ALS, like with many other disorders of the nervous system, is not cell autonomous, that is, initiated by and affecting only one cell type. Furthermore, in ALS both central and peripheral nervous system components are affected by the disease. The disease has been referred to as a dying back phenomena suggesting that initial pathology begins at the neuromuscular junction (NMJ). On the other hand, initial pathology has also been reported to occur in the cell body.

Further characterization of pathological events that occur centrally and peripherally coincident with initial denervation may provide insight into disease onset, help in the discovery of presymptomatic diagnostic disease markers, and identify novel therapeutic targets. In this study, we examined ultrastructual examination of both central and peripheral components of the neuromuscular system in the SOD1^G93A^ mouse model of ALS and related these alterations with motor dysfunction, gait alterations, and muscle weakness. Our results provide insight into the earliest pathological and motor events in this model that can serve as a framework for guiding future research and development of new therapeutic avenues that target these early events.

## Methods

Please see accompanying article (doi: 10.1002/brb3.143) for detailed Materials and Methods.

## Results

### Motoneuron degeneration begins between days 44 and 60

To determine when MN degeneration begins in the SOD1^G93A^ mouse, we evaluated the size and number of MNs.

**Figure 1:**
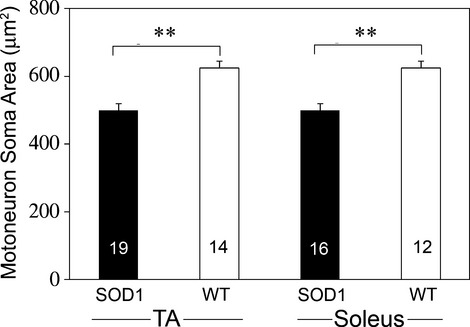
Motoneurons in the TA and soleus motor pools were identified by fluorescent CTB retrograde transport that was injected at P30 and the retrogradely labeled MN soma area was determined at P34. Both SOD1 motor pools were significantly smaller as compared to WT. The number of animals for each condition is indicated in the bars of the graph. **P ≤ 0.01; statistical significance determined by one-way analysis of variance (ANOVA) followed by Bonferroni's multiple comparison test.

At P30, superoxide dismutase 1 (SOD1) MNs had a smaller soma area as compared with those in wild-type (WT) animals. Interestingly, when we evaluated MNs from the TA versus soleus motor pools, we found no difference in the size of MNs between the two pools in either WT or SOD1 spinal cords; however, MNs from both pools were significantly smaller in SOD1 than their WT counterparts (Fig. 1).

**Figure 2:**
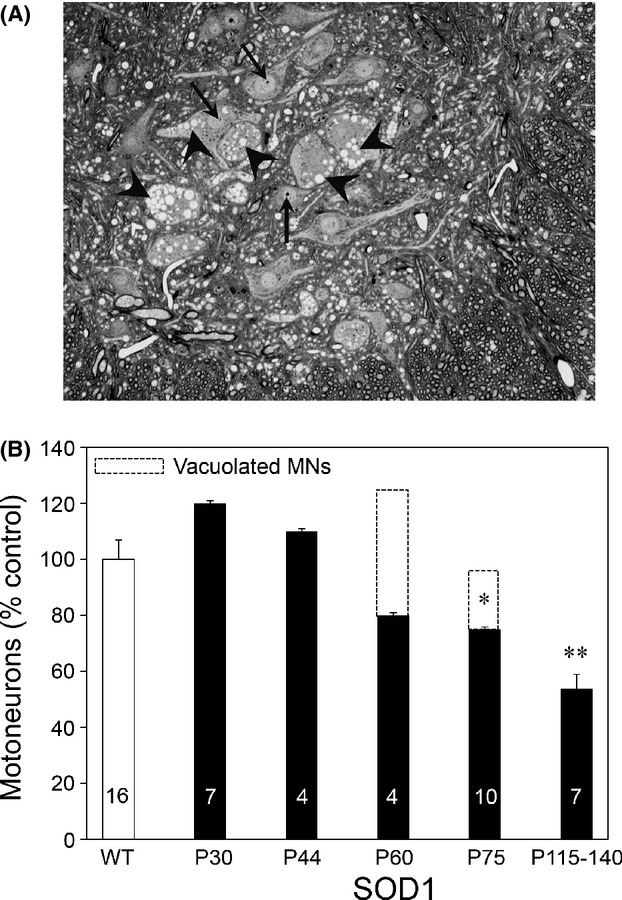
Motoneurons contain numerous cytoplasmic vacuoles that are an early sign of impending degeneration. (A) Photomicrograph of 1 μm section through P60 lateral motor column of SOD1^G93A^ mice. MNs are easily identified by their large size, nucleus, and prominent nucleolus (arrows). Many MNs contain numerous cytoplasmic vacuoles that can be viewed by light microscopy (arrowheads). (B) MNs were counted using well-established criteria that have been previously described (Clarke and Oppenheim 1995). At P60 in the SOD1^G93A^ mouse spinal cord, however, many MNs meet some or all of these criteria even though they contain numerous cytoplasmic vacuoles that are an early sign of impending degeneration viewed by light microscopy. However, if the presumptive degenerating MNs containing cytoplasmic vacuoles are included in cell counts, total numbers are comparable to WT. If vacuolated MNs are excluded from the counts, then there is a 20% decrease at P60 and a 30% decrease at P70 in mutant spinal cords. By P115–140, there are few remaining vacuolated MNs in mutant mice and the number of surviving MNs at this age is reduced by approximately 50%. The number of animals for each condition is indicated in the bars of the graph; *P ≤ 0.05; **P ≤ 0.01; statistical significance determined by *t*-test with Bonferroni correction.

Cell death of MNs in the SOD1^G93A^ mouse has previously been reported to be a late stage event with loss of cell number beginning around day 90 (Chiu et al. 1995; Fischer et al. 2004). Using a well-established criteria for counting MNs (Clarke and Oppenheim 1995) we found that at P60 in the SOD1^G93A^ mouse spinal cord many MNs meet some or all of the criteria for healthy MNs. Many MNs, however, contained numerous cytoplasmic vacuoles. MNs with vacuoles (Fig. 2A) were seen in mutant mice at P60 but not at P44. If we included these obviously pathological cells together with apparent normal, healthy cells in our counts at P60, then total numbers were comparable with WT (Fig. 2B and data not shown). By contrast, if vacuolated MNs were excluded from the counts, then there was a 20% decrease at P60 and a 30% decrease at P75 in mutant spinal cords. By P115–140, there were few remaining vacuolated MNs in mutant mice and the number of surviving MNs was reduced by approximately 50%. The 30% reduction in MNs at P75 reflected the exclusion of vacuolated cells that account for about half of the total loss, and the other half by the complete absence of cell bodies. Between P115 and P140, over 90% of the reduction in cell numbers reflected the actual loss of cell bodies. Taken together, these data indicate that cell death begins between P60 and P75, that cell death is heralded by cytoplasmic vacuolization visible at the light microscope beginning between P44 and P60, and that the total MN loss by end stage is approximately 50% in lumbar spinal cord of mutant mice.

**Figure 3:**
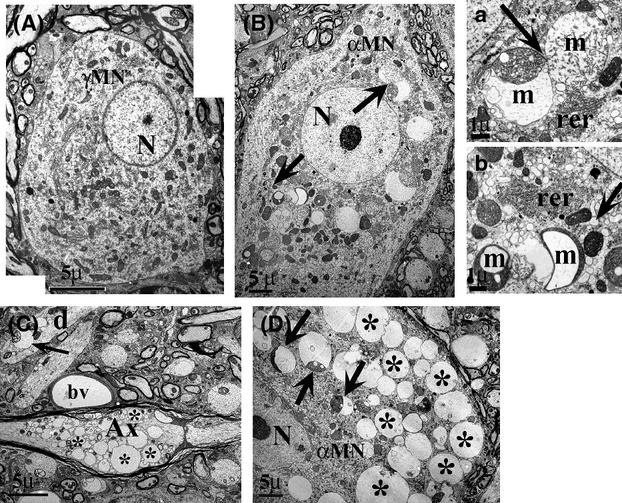
Ultrastructural analysis reveals profound mitochondrial and cytoplasmic pathology by P75. At P75, there is no cytoplasmic pathology in SOD1 γMNs (A; area = 225 μm^2^) but profound pathology in the cytoplasm of αMNs in the same section (B; area = 2414 μm^2^), which can be distinguished on the basis of their size. Arrows in (B) point out areas enlarged in a and b, where one can see swollen and vacuolated mitochondria (m) and accumulated vacuoles; note that rough endoplasmic reticulum (rer) appears normal. (C) There is also extreme disruption in axons at P75 (Ax), which are often engorged with large vacuoles (*). Dendrites d also exhibit similar vacuoles (arrows), in this case resulting from an apparent distension of the outer mitochondrial membrane. (D) Cytoplasm of αMN are filled with huge vacuoles (*) that appear to reflect distended outer mitochondrial membranes as seen more clearly at arrows. Representative images are shown. Three animals of each genotype were examined.

The cytoplasmic organelles responsible for the vacuoles in MNs observed at the light level were examined at the ultrastructual level. Examination of ventral spinal cord at P75 revealed an increased presence of many markedly swollen/vacuolated mitochondria in MNs (Fig. 3). A single, swollen/vacuolated mitochondria often occupied entire portions of dendrites in the neuropil. Another striking feature was the ubiquitous presence of small empty cytoplasmic vacuoles throughout the MN soma. There was no apparent cytoplasmic pathology in putative γMNs (Fig. 3).

### Interneurons also undergo degeneration

**Figure 4:**
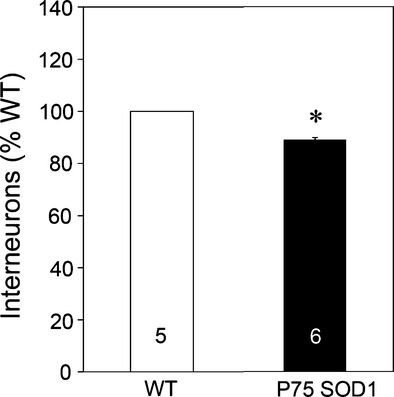
Interneurons also undergo degeneration in SOD1^G93A^ spinal cord. Because we observed mitochondrial swelling and vacuolization in presynaptic terminals on MN soma and dendrites (see Fig. 3), we also counted interneurons to determine if they are decreased in mutant mice. At P75, the number of interneurons in the SOD1 lateral motor column was significantly decreased versus WT. The number of animals for each genotype is indicated in the bars of the graph; *P ≤ 0.05 Statistical significance determined by *t*-test with Bonferroni correction.

While spinal MNs are the main focus of dysfunction and degeneration in ALS, interneurons may also be affected (Eisen 1995; Jiang et al. 2009; Martin and Chang 2012). Because ultrastructural analysis of spinal MNs revealed mitochondrial swelling and vacuolization in presynaptic terminals on their soma and dendrites, we next wanted to determine if there was a degeneration of spinal cord interneurons because they were the source of the majority of these afferent terminals. We found that at P75, the number of interneurons in the lumbar spinal cord was significantly decreased (Fig. 4).

### Ventral roots atrophy, but absolute numbers are not reduced by P75

**Figure 5:**
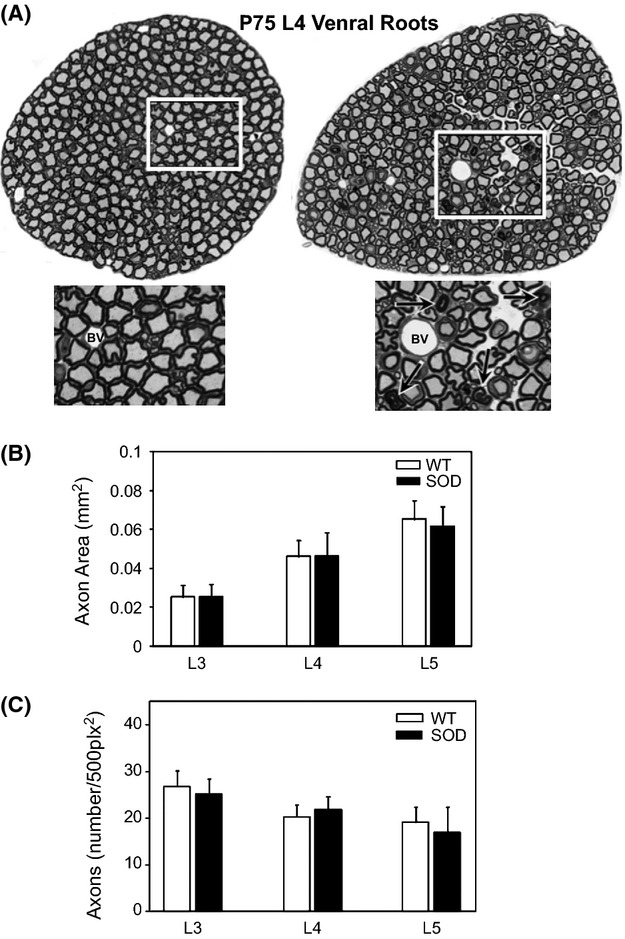
Ventral roots atrophy, but absolute number of axons are not reduced by P75. Ventral root axons from L3, L4, and L5 segments of spinal cord were counted to determine if there was a degeneration of ventral roots associated with the apparent degeneration of MNs and interneurons at day 75. There was no significant difference in the area of (B) or number of (C) axons in SOD1 versus WT ventral roots (*t*-test with Bonferroni correction). Many axons in the SOD1 mouse, however, exhibited alterations indicative of ongoing or impending demyelination/degeneration (see enlarged boxed regions in A). *n* = 6 animals/genotype.

We next wanted to determine if there was a degeneration of ventral roots associated with the apparent degeneration of MNs at day 75. L3, L4, and L5 ventral roots were counted and no significant difference was observed between SOD1 and WT. While there was no change in the absolute number of ventral root axons, many axons in the SOD1 mouse exhibited alterations indicative of ongoing or impending demyelination and degeneration (Fig. 5).

### Early loss of intramuscular axons and muscle denervation

In a previous study, we identified initial denervation of the medial gastrocnemius muscle (MG) by postnatal day 25 (P25; Gould et al. 2006). The MG is a mixed muscle containing both fast and slow fibers. Here, we examined the tibialis anterior (TA) muscle composed of only fast fibers and the soleus muscle composed of slow fibers. In the TA muscle, the compartment located adjacent to the skin (outer) contains predominantly type IIB fibers, whereas the muscle compartment adjacent to the bone (inner) contains a mix of type IIA and IIB fibers. The outer compartment undergoes denervation before the inner compartment (Pun et al. 2006).

**Figure 6:**
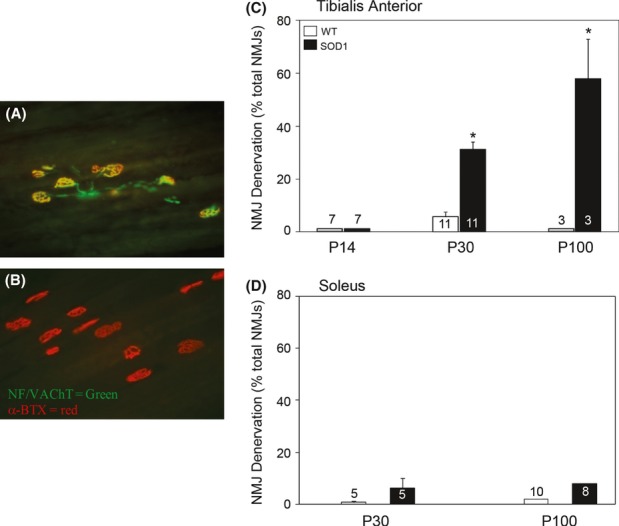
Denervation in SOD1^G93A^ FF muscles (TA) occurs between P14 and 30. (A and B) Photomicrographs are shown of P30 TA muscles from WT (A) and SOD1^G93A^ (B) mice. Alexa fluor 555-α-BTX was used to identify postsynaptic terminals (red) and antibodies to VAChT and neurofilament (NF) were used to identify presynaptic terminals and axons, respectively, and were visualized with Alexa fluor 488 secondary antibodies (green). NMJs that exhibited an overlap of red and green were considered innervated, while those that exhibited only α-BTX expression were considered denervated. Note the obvious denervation in the SOD1^G93A^ TA in B. (C) Muscle innervation was examined in the P14, P30, and P100 TA in SOD1^G93A^ and WT mice. While significant denervation occurs at P30, there is no denervation at P14, but by P100, 70% of TA NMJs were denervated. The results are presented as % denervated of total NMJs/muscle (mean ± SEM). (D) Muscle innervation was examined in the P30 and P100 soleus in SOD1^G93A^ and WT mice. There was little denervation in SOD1 animals even at P100. The number of animals for each condition is indicated in the bars of the graph; *P ≤ 0.05 as compared to WT as determined by unpaired *T*-test.

**Figure 7:**
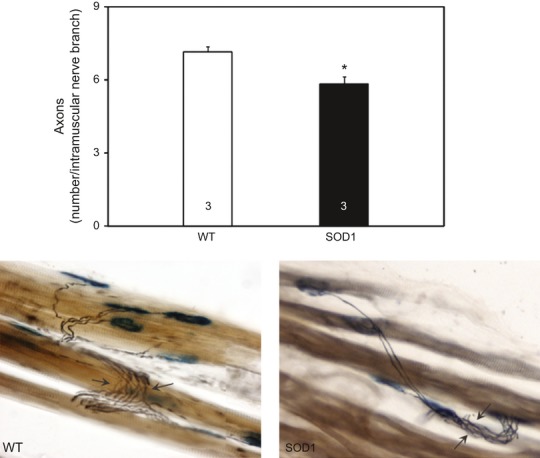
Loss of axons in intramuscular nerve branches in the TA muscle of SOD1 mutant mice at P30. Intramuscular nerve branches (black axon bundles) and postsynaptic sites (acetylcholine esterase, blue) were labeled, and axons in intramuscular nerve branches (arrows) that could be followed to NMJs were counted as described in the methods. A total of 22–25 nerve branches were counted in each muscle. Results are expressed as mean number of axons in intramuscular nerve branch ± SEM. The number of animals for each condition is indicated in the bars of the graph; *P ≤ 0.05 as compared to WT as determined by unpaired *T*-test.

A denervated NMJ was one that exhibited α-bungarotoxin (α-BTX) postsynaptically and the absence of vesicular acetylcholine transporter (VAChT) in the presynaptic terminal (Fig. 6A and B). Denervation of TA began after P14 (0% denervation) but before P30 (40% denervation), and continued with disease progression (Fig. 6C). On the other hand, the soleus showed no denervation at P30. There was a slight increase in denervation above WT at late postsymptomatic stages; however, the differences were not statistically significant (Fig. 6D). We also examined adjacent sections in which presynaptic terminals were identified using antibodies to SV2 or synaptophysin. This analysis yielded almost identical results as those that used the antibody to VAChT suggesting that the absence of VAChT indicated denervation and not decreased expression of the antigen (data not shown). We also found a decrease in the number of silver esterase-labeled axons in intramuscular nerve branches in the P30 SOD1 TA muscle versus WT mice (Fig. 7).

**Figure 8:**
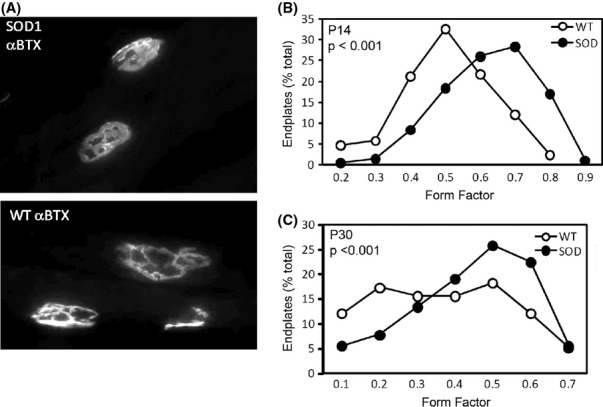
Endplate morphometry (form factor) was assessed for NMJs (postsynaptic a-BTX-positive endplates; A) in tibialis anterior of wild-type and SOD1 mice at P14 (B) and P30 (C). In both cases there is a shift to the right that indicates that in SOD1 mice, endplates have a less elongated shape than in WT animals. There was a significant difference between SOD1 and WT TA at P30 (*P* ≤ 0.01) and at P14 (*P* ≤ 0.001). There was no difference between SOD1 and WT soleus (not shown). *n* = 4 animals/genotype/age; 90 NMJs/muscle/animal were examined. Statistical significance was determined by Mann–Whitney test.

Although there was no NMJ denervation in the P14 TA muscle in SOD1 mice, we next asked if there were other changes in the NMJ that might portend future denervation. We therefore determined the form factor for the postsynaptic terminal (Brunet et al. 2007). Form factor is 4ϕ area/perimeter and is used as an indicator of the degree of roundness of a structure; values closer to one indicate a more spherical structure. The form factor for the majority of P14 SOD1 NMJs was closer to one than those from WT animals (Fig. 8). A similar result was also observed at P30. The differences in shape of the NMJ between WT and SOD1 mice may indicate alterations in development of the NMJ or impending denervation in SOD1 mice prior to terminal fragmentation (Schaefer et al. 2005; Valdez et al. 2010), and are in agreement with apparent shape change reported previously in SOD1 NMJs that will soon (in days) undergo denervation (see Fig. 5 in Pun et al. 2006). We also examined the shape of the NMJs in the soleus muscle at P30 and found no difference between SOD1 and WT (data not shown). Together, these results suggest that apparent signs of impending denervation can be detected by P14 in the TA muscle.

### Axonal transport

**Figure 9:**
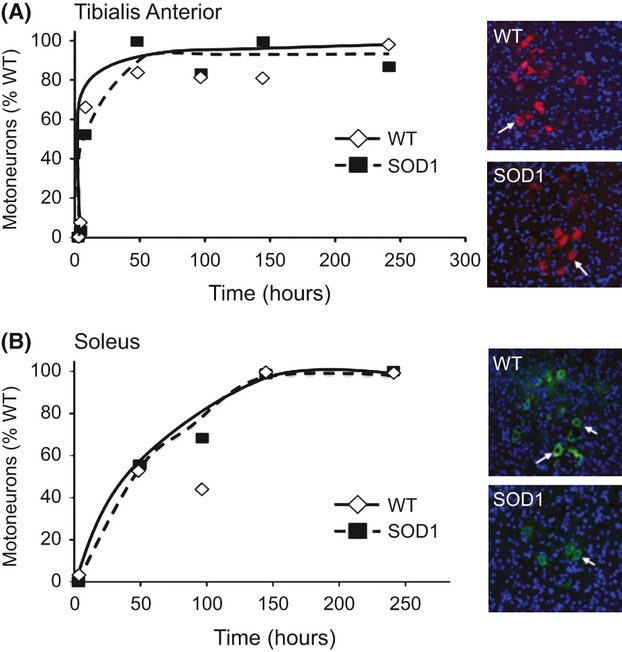
Retrograde transport in MNs innervating the tibialis anterior (TA) and soleus muscles was examined in mice at P20 using Alexa Fluor®555 and ®488 conjugated with cholera toxin B-subunit (CTB), respectively. (A) There was no statistically significant difference in retrograde transport in MNs innervating the TA (A) or soleus (B) between SOD1^G93A^ and WT mice. Individual time points are shown with symbols and a best-fit line was drawn. Three to eight animals/genotype were examined at each time point. Statistical significance was determined by ANOVA followed by Tukey–Kramer post hoc test.

Deficits in axonal transport are reported to contribute to pathology in neurodegenerative diseases (reviewed in Morfini et al. 2009; Sau et al. 2011). In ALS mouse models, deficits in both retrograde and anterograde transport are reported to be early events in disease pathology (Williamson and Cleveland 1999; Bilsland et al. 2010). We injected the TA or soleus muscles of P20 mice with Alexa fluor-conjugated Cholera toxin subunit B (CTB). We found that at this age (P20) there was no difference in the rate of retrograde transport in either TA or soleus innervating axons in SOD1 versus WT mice (Fig. 9). These results suggest that alterations in retrograde transport are not associated with initial muscle denervation.

### Ultrastructual analysis of the presynaptic terminal at the NMJ in SOD1 mice

**Figure 10:**
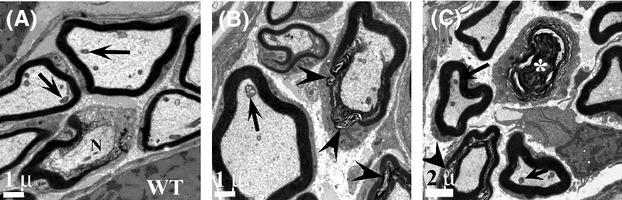
At P30, mitochondria (arrows) in intramuscular axons of all SOD1 muscle types examined had swollen mitochondria as compared with WT (A), although this effect most prominent in outer TA (B). A node of Ranvier (N) can be seen in (A), while myelin sheath aberrations are in SOD1 material (arrowheads in B). (C) On P53, axons in TA intramuscular axons exhibit frank degeneration (*) as well as myelin sheath aberrations. Representative images are shown. Four animals of each genotype were examined.

Given the presence of vacuolated mitochondria and small, clear cytoplasmic vacuoles in MNs at the beginning of degeneration, we next asked if similar pathological changes are seen in intramuscular axons in the P30 TA (inner and outer compartments) and soleus muscle at the time of early muscle denervation. Larger and more vacuolated mitochondria were observed in axons innervating both compartments of the mutant versus WT TA (Fig. 10). The occurrence of altered mitochondria was increased in TA muscle axons, but mitochondrial pathology was also occasionally observed in the soleus axons (not shown). Mitochondria in Schwann cells and muscle, including the postsynaptic region of the NMJ did not show any sign of pathology. There was apparent demyelination of axons in the outer portion of the TA (Fig. 10).

**Figure 11:**
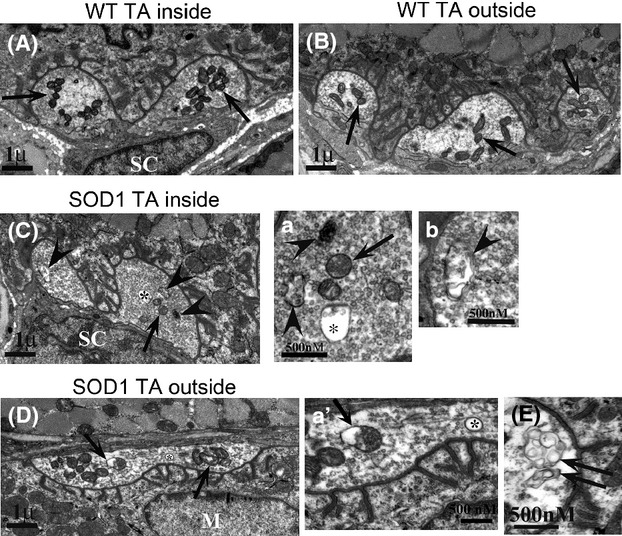
(A and B) Normal NMJ appearance in the inner TA (types IIa and IIb) and outer TA (primarily type IIb), respectively in P30 animals. Arrows point to normal compact terminal mitochondria. (C) In SOD1 animals, alterations in inner TA, enlarged in a and b, while (D) shows outer TA, enlarged in a'. Asterisks in a and a' are inside of large vacuoles (>100 nm diameter), a common feature in SOD1 animals. Arrows point out swollen mitochondria, and arrowheads point out abnormalities that appear autophagic-like. Double arrows in (E) point to abnormal whorls in a terminal from the outside portion of the TA. Overall, abnormalities of all types were seen 2–3 times more frequently in SOD1 than WT. Representative images are shown. Five animals of each genotype were examined.

**Figure 12:**
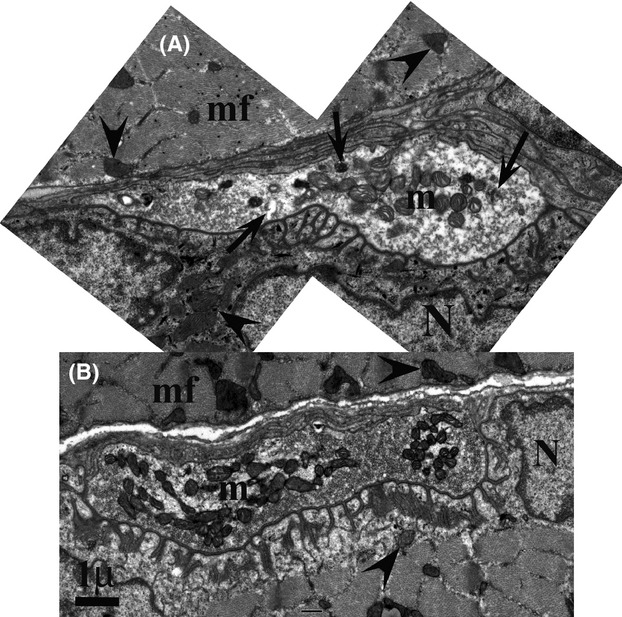
P30 SOD1 soleus NMJ exhibit slightly swollen mitochondria in the terminal (m in A) compared with WT (B), although mitochondria in the muscle fibers (mf) and sarcoplasm (arrowheads) remain normal. Numerous junctional folds are present. Note the occasional abnormality (arrows) in A. Magnification marker = 1 μm for both A and B; N = nucleus of muscle cell. Representative images are shown. Five animals of each genotype were examined.

In the presynaptic terminal of P30 SOD1 TA, 50% of NMJs had aberrations in mitochondria and/or degenerative inclusions (Fig. 11). These morphological changes were consistently observed in NMJs in both inner and outer compartments of the muscle. Similar morphological changes were also observed in SOD1 soleus presynaptic terminals, but to a lesser extent than observed in the TA (Fig. 12).

**Figure 13:**
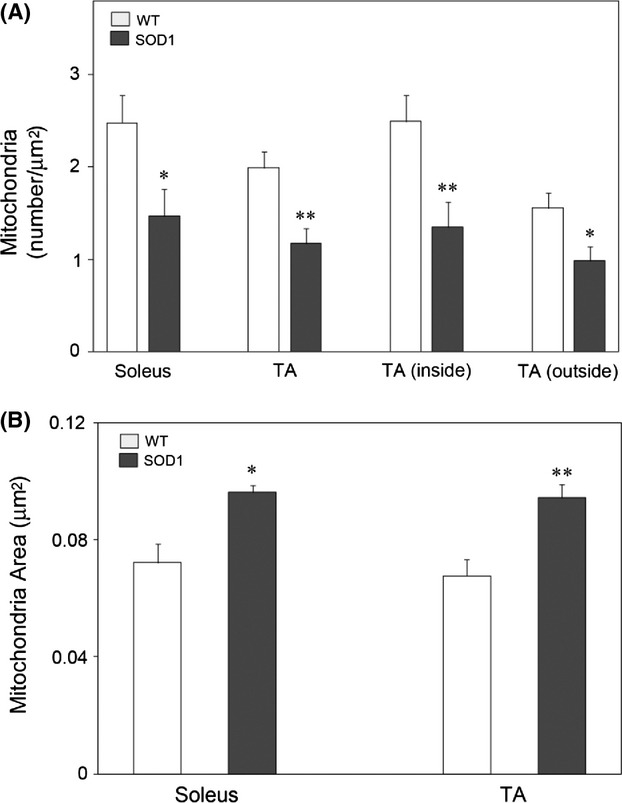
Fewer, but larger mitochondria are present in presynaptic NMJs from SOD1 animals versus WT. The number and area of mitochondria was determined as described in Materials and Methods. (A) The absolute number of mitochondria is decreased in SOD1 versus WT presynaptic NMJs. (B) While there is a decrease in the number, there is an increase in the area of mitochondria in P30 SOD1 versus WT. Results are expressed as mean ± SEM. Thirty NMJs/animal were examined (*n* = 5 animals/group). Significant difference between groups was determined using unpaired *t*-tests. *P ≤ 0.05; **P ≤ 0.01.

To confirm that there were differences in the size of SOD1 mitochondria versus WT, size and area of mitochondria in the NMJ presynaptic terminals in the TA and soleus muscles were measured. In SOD1 animals, the number of mitochondria in both TA and soleus presynaptic terminals was reduced as compared with WT (Fig. 13A). The decrease in number occurred in NMJs in both the inner and outer compartments of the TA muscles. By contrast, the area of individual mitochondria was significantly increased in both TA and soleus presynaptic terminals of SOD1 animals (Fig. 13B). Together these results suggest alterations in mitochondrial fission and/or fusion in the presynaptic terminals.

We also evaluated several additional features of the NMJs of TA and soleus muscles at P30 (Table 1). We found that the junctional fold length was shorter in NMJs in the outer compartment of the SOD1 TA as compared with WT, but no significant difference was found for the same measure in the inner compartment or in the soleus muscle. Interestingly, the decrease in junctional fold length has also been observed in the SMAΔ 7 mouse model and is thought to represent a developmental delay in NMJ formation (Lee et al. 2011). There were no differences in the diameter or number of junctional folds. There was also an apparent reduction in the number of docked vesicles/μm active zone in both compartments of the TA and in soleus muscles in SOD1 animals versus WT, although this difference did not reach statistical significance. There was no difference in the total number of vesicles/μm^2^ in the presynaptic terminal between WT versus SOD1 mice. A number of additional aberrations in SOD1 NMJs, including whorls, empty vacuoles >100 nm in diameter, and autophagic-like bodies (Fig. 11), were approximately two- to fourfold times more common than in WT, indicating early pathology.

**Table 1:** Characterization of NMJ presynaptic terminal

Area	WT TA (o)	SOD TA (o)	WT TA (i)	SOD TA (i)	WT Soleus	SOD Soleus
#Dock vesicles/μm AZ	5.11 ± 0.65	4.91 ± 0.65	5.67 ± 0.97	4.62 ± 0.96	5.99 ± 0.63	4.55 ± 0.62
#Vesicles/μm^2^ terminal	55.00 ± 7.60	55.83 ± 7.58	54.94 ± 7.59	53.96 ± 7.47	65.65 ± 6.80	54.77 ± 6.72
#Junctional folds/μm AZ	1.60 ± 0.10	1.60 ± 0.10	1.45 ± 0.14	1.27 ± 0.13	1.12 ± 0.11	0.95 ± 0.11
Junctional fold length (nm)	**683.48 ± 22.58**	**598.26 ± 21.93**	617.28 ± 36.08	562.52 ± 32.82	595.13 ± 32.98	553.27 ± 31.78
Junctional fold diameter (nm)	65.24 ± 2.90	67.60 ± 2.88	72.014 ± 4.46	65.26 ± 4.27	79.06 ± 4.43	73.02 ± 4.30

Results expressed as mean + SEM; numbers in bold have a statistical significant difference SOD1 versus WT (*P* ≤ 0.01). Statistical significance was determined by repeated measures comparisons to assess differences between continuous study outcomes by group. At least 26 NMJs were examined from each muscle in each group (SOD1 vs. WT) with *n* = 4 animals/group. i, inner; o, outer.

**Figure 14:**
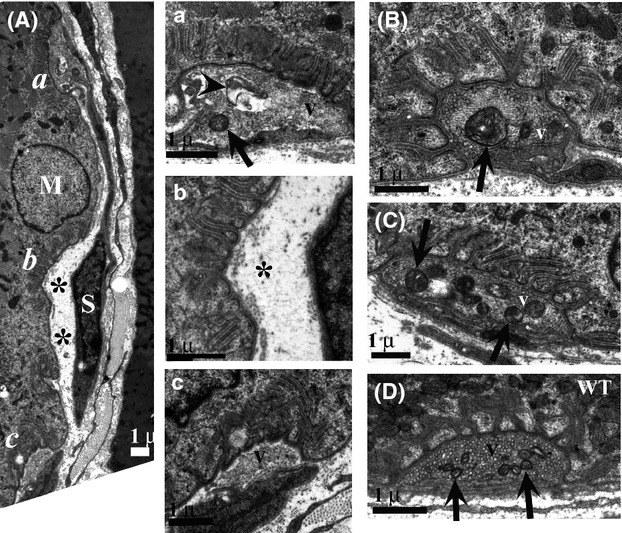
(A) On P53 presynaptic terminals of NMJs in the outer component of the TA show advanced degeneration. Three areas of the terminal are enlarged in a, b, and c: (a) illustrates a region of the presynaptic terminal that contains vesicles (v), but has a large autophagic-like body (arrowhead); (b) illustrates a region of the same terminal that is completely devoid of vesicles (*); (c) illustrates another portion of the terminal that contains vesicles that appear normal. (B) Other presynaptic terminals in this region have a range of appearances and include some that are relatively normal except for swollen mitochondria (arrows). (C) Presynaptic terminals in the inner portion of the TA of SOD1 animals show less severe effects, and often appear normal except for swollen mitochondria (arrows). (D) A NMJ from the outer portion of the TA from a WT animal shows normal morphology of synaptic vesicles (v) and mitochondria (arrows). *n* = 4 animals for SOD1 and four animals for WT.

We also examined ultrastructure of intramuscular axons and presynaptic terminals in the TA muscle on P53 when denervation of NMJs is progressing. The outside component of the TA muscle (adjacent to the skin) is composed of type IIB fibers and NMJs in this region are reported to be devoid of synaptic vesicles by this age (Pun et al. 2006). Intramuscular axons in the outside (skin) component of the TA of SOD1 animals showed signs of frank degeneration (Fig. 14). With the exception of having enlarged mitochondria some presynaptic terminals in the outside portion of the TA had an appearance similar to WT animals; however, many exhibited signs of more advanced degeneration that at P30 (Fig. 14). We found individual NMJs with both normal and abnormal nerve–muscle contacts, including some with an absence of synaptic vesicles (Fig. 14).

**Figure 15:**
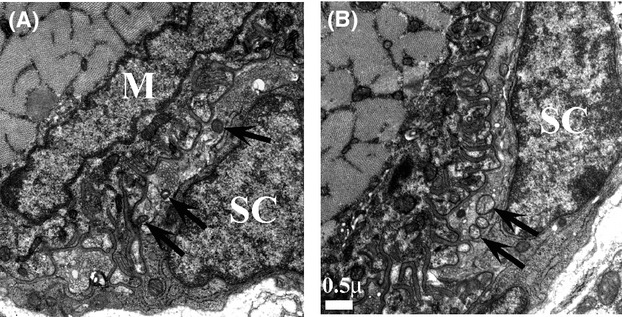
At P7, SOD1 TA NMJ often show slightly swollen mitochondria as compared with WT (A = WT; B = SOD1; arrows). The NMJ presynaptic terminal is shaded gold and lies between a terminal Schwann cell (SC) and the postsynaptic muscle (M). Representative images are shown. Three animals of each genotype were examined.

Quantification of NMJ denervation in the TA muscle at P14 failed to reveal any differences between WT and mutant mice, indicating that the onset of denervation of NMJs in type IIB muscle occurs between P14 and P30. However, abnormal mitochondria were observed in a subset of terminals at the NMJ (30%) in the mutant TA at P14 indicating that mitochondrial changes precede the onset of denervation (not shown). Similar changes were also observed as early as P7 in the SOD1 TA muscle (Fig. 15). These results further suggest presynaptic terminal mitochondrial abnormalities precede NMJ denervation.

### Mitochondrial abnormalities and cytoplasmic vacuoles are prominent in the spinal cord

**Figure 16:**
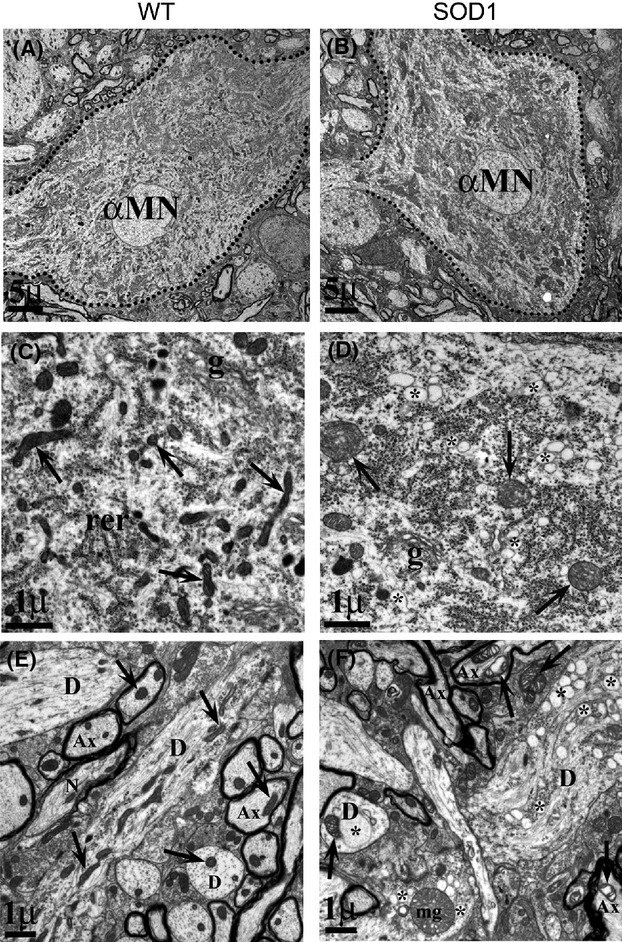
Enlarged mitochondria and cytoplasmic vacuoles are observed in P30 SOD1 MNs. At P30, αMNs have a similar appearance in both WT (A) and SOD1 (B) animals. However, upon closer inspection, mitochondria (arrows in C–F) are larger in SOD1 (D) as compared with WT (C). There is also an accumulation of numerous small vacuoles in the cytoplasm of SOD1 MNs (* in D and F). In the neuropil, these effects are more striking, as seen in SOD1 (F) versus WT (E). Axons (Ax) and dendrites (D) contain quite swollen mitochondria (arrows) and larger, more numerous vacuoles (*), as well as mega-mitochondria (mg). We examined ultrastructural changes in MNs in the L3–L4 segments of the spinal cord at day 30 of SOD1^G93A^ mice (*n* = 5, total of 38 MNs) and compared these findings with age-matched wild-type (WT) animals (*n* = 5, total of 48 MNs).

We next wanted to determine whether similar changes in the ultrastructure of NMJ mitochondria were also observed in MN soma and dendrites in the spinal cord at the time of early muscle denervation. At P30, MNs contained swollen or vacuolated mitochondria as well as mega-mitochondria that were observed at the electron microscope level, but not at light microscope level as seen at P60 (Fig. 16 vs. Fig. 2). In swollen or vacuolated mitochondria, space between the inner and outer membranes appeared to be enlarged, whereas mega-mitochondria were characterized as significantly enlarged mitochondria with an apparent normal internal structure. Interestingly, presynaptic terminals on MNs also possessed morphologically abnormal mitochondria. These features were observed in all α-MNs examined, whereas the terminals on γ-MNs appeared normal. Furthermore, while α-MNs exhibited these morphological abnormalities, the extent was not the same in all cells, and often the affected cells included MNs outside the putative TA MN pool.

There was also an accumulation of small empty vacuoles in cytoplasm of MNs in SOD1^G93A^ mice (Fig. 16D). These vacuoles were identical in appearance to those observed at P75, but were far fewer in number. Although the source of these vacuoles is not clear, they were often observed close to endoplasmic reticulum (ER) and *cis*- or *trans*-Golgi elements and less often also observed in association with mitochondria. However, these vacuoles were rarely observed in axons and were not observed in the presynaptic terminal of the NMJs, lending further support to their possible derivation from ER or Golgi.

**Figure 17:**
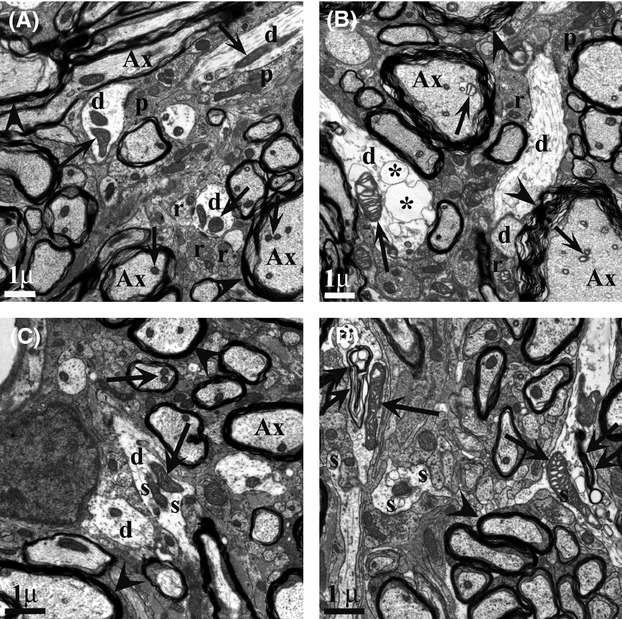
Distal dendrites in SOD1 MNs exhibit vacuolated mitochondria and large cytoplasmic vacuoles, and axons have fragmented myelin sheaths. Synapse types on MN distal dendrites (d) at P30 in white matter adjacent to VH showed a decrease in type I (r) synapses similar to that seen for MNs, with no significant decrease in type II (p). Note distension of mitochondrial cristae (arrows) in SOD1 (B) compared with WT (A) in both axons (Ax) and dendrites, as well as poor condition of myelin sheath (arrowheads) and presence of large vacuoles (*) in SOD1 (*n* = 5 animals each, WT and SOD1). Double arrows in D point to large autophagic-like bodies in dendrites. For distal dendrites, using the same levels as used for MN soma evaluation in Figure 16, 20–30 dendrites with synapses from each animal used above were located in the ventro-lateral white matter and photographed at 16,000×. Only synapses with a clear synaptic density and presynaptic vesicles were scored.

We also observed mitochondrial abnormalities and large cytoplasmic vacuoles in both proximal and distal dendrites of P30 α-MNs (Figs. 16F and 17). Distal MN dendrites that extend into ventral and ventral–lateral white matter exhibited the most profound morphological abnormalities at P30; these mitochondria had swollen and vacuolated spaces between the internal membrane and substantially larger cytoplasmic vacuoles than those observed in the soma (Fig. 17). The morphology of these vacuoles is similar to that observed in heart or liver mega-mitochondria following chronic exposure to a hypotonic solution (Wakabayashi 2002).

**Figure 18:**
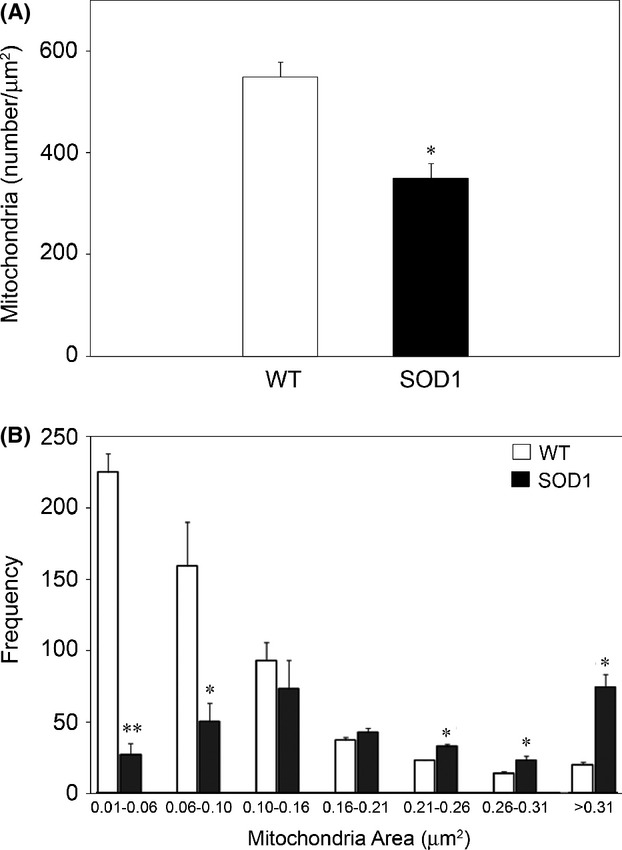
Fewer, but larger mitochondria are present in MNs from SOD1 animals versus WT. The number and area of mitochondria was determined as described in Materials and Methods. (A) There is a decrease in the number of mitochondria in P30 SOD1 MNs versus WT MNs. (B) While the number of mitochondria was decreased in SOD1 MNs, there was an increase in the number of larger sized of mitochondria in SOD1 versus WT MNs (B). Mitochondria from the MNs examined in Figure 16 were examined (*n* = 5 animals/group for a total of 38 MNs in SOD1 and 48 MNs in WT). Significant difference between groups was determined using unpaired *t*-tests (*P < 0.05; **P < 0.001).

Because swollen and vacuolated mitochondria are a prominent feature of pathogenesis at P30, we next quantified the extent of changes in mitochondrial size and found that while as noted above the number of mitochondria in MNs was reduced, the size was substantially greater in SOD1 animals than in WT mice (Fig. 18). To determine if ultrastructural changes in SOD1 mitochondria is associated with altered function, mitochondria were isolated from SOD1 and WT lumbar spinal cords. Mitochondria from SOD1 animals had a 30% reduction in membrane potential (WT = 1.54 fluorescent units [FU]/55 μg mitochondria protein vs. SOD1 = 1.10 FU/55 μg mitochondria protein). ATP content was 1.5 times higher in WT versus SOD1 mitochondria (0.055 μmol/L WT vs. 0.036 μmol/L SOD1), and ATP generation was reduced by 35% in SOD1 versus WT animals (0.079 μmol/L per mg mitochondria protein WT vs. 0.051 μmol/L per mg mitochondria protein SOD1).

### Alterations in synaptic input

**Figure 19:**
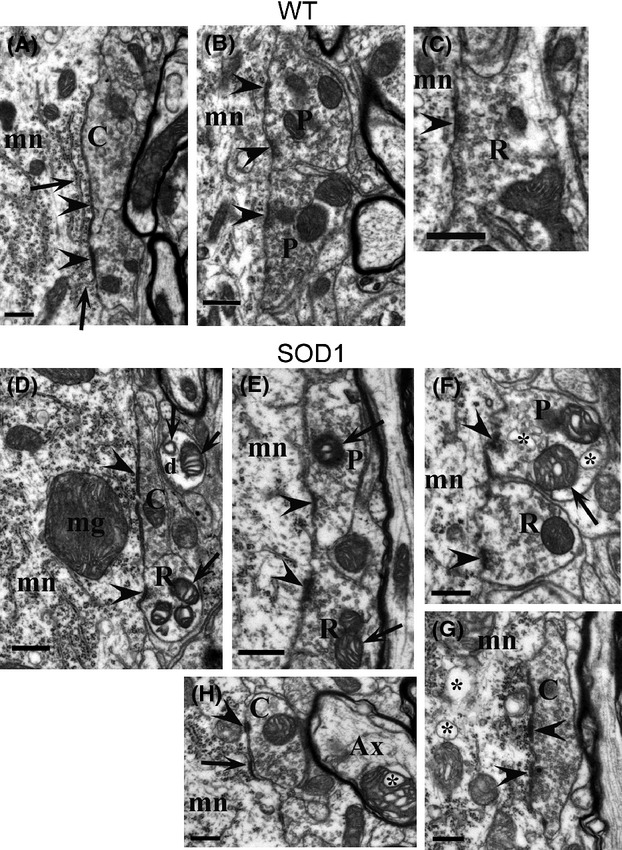
Illustrations of synapse types in WT (A–C) and SOD1 (D–G) MNs. C-terminals, which are restricted to αMNs and are characterized by subsynaptic cisterns and organelles (arrows in A, G) and contain irregularly shaped and densely packed vesicles, showed classic appearance in both WT (A) and SOD1 (D) mice; however, some C-terminals in SOD1 spinal cord were atypical, with smaller areas and less distinctive vesicle density (G, H). Typical type I (R) and type II (P) synapses were observed on WT (B, C) and SOD1 (E, F) MNs, although terminals in SOD1 often exhibited swollen mitochondria (arrows, E and F, and mega-mitochondria, mg) and vacuoles (*) both pre- and postsynaptically. Arrowheads point to synaptic densities; mn, motoneuron; mag marker = 500 nm.

**Figure 20:**
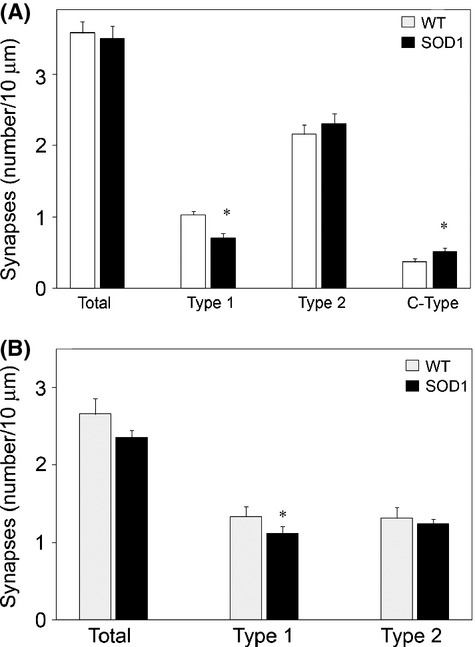
There is a decrease in type I and increase in C-type synapses on SOD1 MNs. Synapses meeting ultrastructure criteria for type I, type II or C-type were counted on MN soma (A) and distal dendrites (B). The total number of synapses was not different between WT and SOD1; however, there was a significant decrease in type I and increase in C-type axo-somatic synapses on SOD1 versus WT MNs. There was a decrease in the number of total axo-dendritic synapses on distal dendrites. There was a significant decrease of type I synapses on distal dendrites. Axo-somatic synapses on the MNs examined in Figure 16 were examined (*n* = 5 animals/group for a total of 38 MNs in SOD1 and 48 MNs in WT). For distal dendrites, using the same levels as use for MN evaluation, 20–30 dendrites with synapses from each animal were located in the ventro-lateral white matter. *P < 0.05; a mixed models approach was initially used to identify differences between WT and SOD1 while accounting for the repeated measures within each mouse and correlation between measures within each mouse. Statistical difference was determined using a least square means table.

Alterations in afferent signaling to MNs have been proposed to contribute to pathology in ALS. We therefore determined if there were differences in number or type of MN afferent synapses (Fig. 19). At P30, there was no significant change in the total number of axo-somatic synapses on MNs or of type II “inhibitory” synapses (Fig. 20A). There was, however, a significant decrease in type I excitatory synapses and a significant increase in C-terminals. The increase in C-terminals was also confirmed by counting VAChT immunopositive synapses onto MN soma. SOD1 had a 28% increase in VAChT-positive synapses that was statistically significantly different from WT (*P* ≤ 0.05; data not shown). There was also a reduction in total numbers of axo-dendritic synapses on the distal MN dendrites (Table 2), and a significant decrease in the number of type I axo-dendritic synapses at P30 (Fig. 20B). Between P14 and P30 there was a significant increase in the number of MN axo-dendritic synapses in the ventral horn white matter of WT animals (Table 2) whereas SOD1 animals failed to exhibit a similar increase in these synapses between P14 and 30. At P14 SOD1 mice exhibited a significant increase in the number of synapses compared with WT. Together these results suggest that although MN afferent innervation on distal dendrites may increase developmentally in both WT and mutant mice the increase is less than that observed in WT at P30.

**Table 2:** Number of axo-dendritic synapses/10 μm membrane on distal MN dendrites

	WT	SOD1	WT versus SOD1
P14	1.732 + 0.163	2.234 + 0.260	*P* = 0.0473
P30	2.662 + 0.446	2.003 + 0.203	*P* = 0.0166
P14 versus P30	*P* = 0.0147	*P* = 0.2036	

Results expressed as mean ± SD. P14: *n* = 3 each genotype; P30: *n* = 6 each genotype.

### Ventral horn white matter is altered in SOD1^G93A^ mice at P14 and P30

A noted above, there was a significant increase in the total number of axo-dendritic synapses in the ventral horn white matter at P14 in the SOD1 animals (Table 2). However, at this age, it was not possible to unambiguously categorize synapses as excitatory versus inhibitory by morphological criteria. The increase in the number of synapses may reflect the significantly increased number, but smaller diameters of SOD1 axons in the white matter as compared with WT littermates at P14 (Table 3; Fig. 17). At this same age, there was also a significant increase in the number of glial cells in SOD1 versus WT but no apparent difference in the size (width) of the white matter. At P30, the number of axons in the SOD1 white matter although elevated, did not differ significantly from WT, and although there was no difference in the size of the axons at this age, the size (width) of white matter was significantly reduced in P30 SOD1 versus WT animals.

**Table 3:** Ventral horn white matter axons and glia

	Axon number/mm^2^	Axon area (μm^2^)	White matter width (mm)	Glia/mm^2^
P14				
WT	1249.7 ± 53.797	1.2497 ± 0.1454	147.53 ± 12.792	18.667 ± 1.528
SOD1	1427.1 ± 38.424	1.1055 ± 0.0918	135.1 ± 8.129	25.4 ± 1.342
*P*	0.0015	0.028	0.1373	0.0006
P30				
WT	1094.167 ± 126.34	1.7237 ± 0.1069	237.45 ± 27.161	11.1667 ± 3.430
SOD1	1193.333 ± 113.9	1.6577 ± 0.1574	199.867 ± 6.484	11.8333 ± 1.472
*P*	0.0558	0.242	0.0151	0.6711

Results expressed as mean ± SD. P14: *n* = 3 each genotype; P30: *n* = 6 each genotype.

### Morphological abnormalities in SOD1^G93A^ MNs are detected as early as the first postnatal week

**Figure 21:**
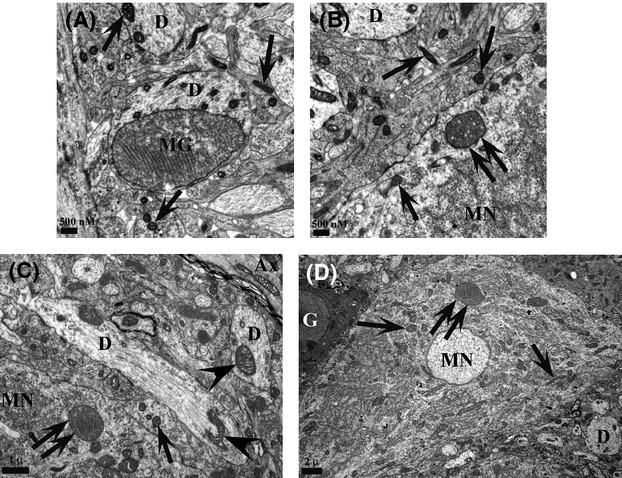
Mega-mitochondria are prominent at P7 and P14. (A and B) Images from the P7 ventral horn of SOD1^G93A^ mice show mega-mitochondria (MG, double arrows) in both dendrites (D) and MN soma (MN). Single arrows indicate normal mitochondria. (C and D) Mega-mitochondria (MG), slightly swollen mitochondria (arrowheads) and normal mitochondria (single arrows) are present in dendrites and MNs of P14 mutant spinal cords. Mega-mitochondria were not observed in WT spinal cord. Representative images are shown. Three animals/genotype/time point were examined.

We next asked how early these morphological abnormalities occurred in MNs in the spinal cord of mutant mice. As early as P7 there was a slight swelling of mitochondria as compared with WT MNs, and mega-mitochondria were frequently observed (Fig. 21), but the small cytoplasmic vacuoles were not present. Similar results were also seen at P14, although mitochondrial swelling was more prominent compared with P7, but less than observed at P30, and the small cytoplasmic vacuoles described above were first observed.

### Ultrastructural analysis of glial cells does not reveal abnormalities observed in neurons

**Figure 22:**
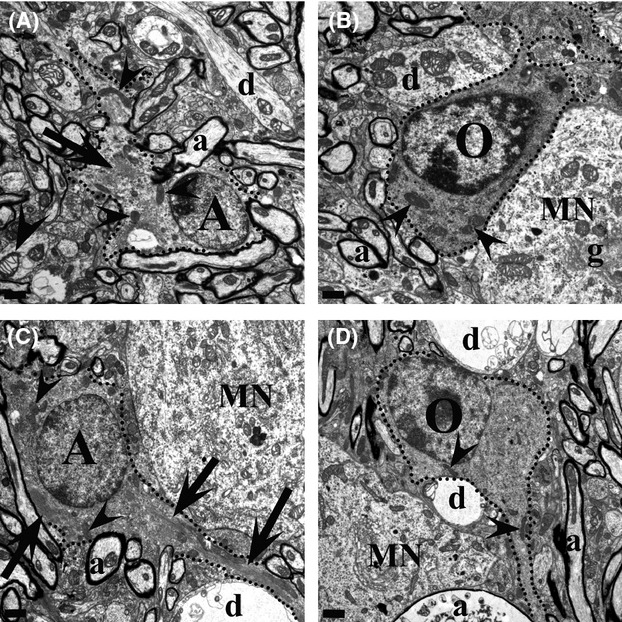
Glia cells do not exhibit cytoplasmic abnormalities. (A and B) At P30, both astrocytes (A) and oligodendrocytes (O) appear to have normal mitochondria (arrowheads) and cytoplasm, although swollen mitochondria can be seen in surrounding neuropil. (C and D) At P110 (*n* = 5 animals/genotype), an astrocyte (A) still shows normal ultrastructural elements, though increased filament bundles (arrows) indicate activation. The oligodendrocyte (O) in D likewise has normal elements and mitochondria (arrowheads). Although not quantified, at P110, oligodendrocytes appear to be more abundant. In both C and D, some ghost-like remnants of dendrites (d) and axons (a) are present. MN, motoneuron; scale bar = 1 μm.

We also examined glial cells in lateral motor column at different ages (Fig. 22). The abnormalities and morphological changes observed in MNs were not observed in astrocytes, oligodendrocytes, or microglia (not shown) at P7, 14, or 30. While there were no morphological abnormalities, there was an increase in the number of glial in the white matter of the ventral lumbar spinal cord at P14 (Table 3). At more advanced ages (P75 or P100), astrocytes exhibited increased expression of filaments presumably associated with their activation, but we never observed any mitochondrial vacuolization or small cytoplasmic vacuoles (Fig. 22). Additionally, oligodendrocytes always appeared normal and although they appear to be more numerous in material from P75 and P100 spinal cords, this putative difference was not quantified.

### Initial NMJ denervation is associated with motor deficits

**Figure 23:**
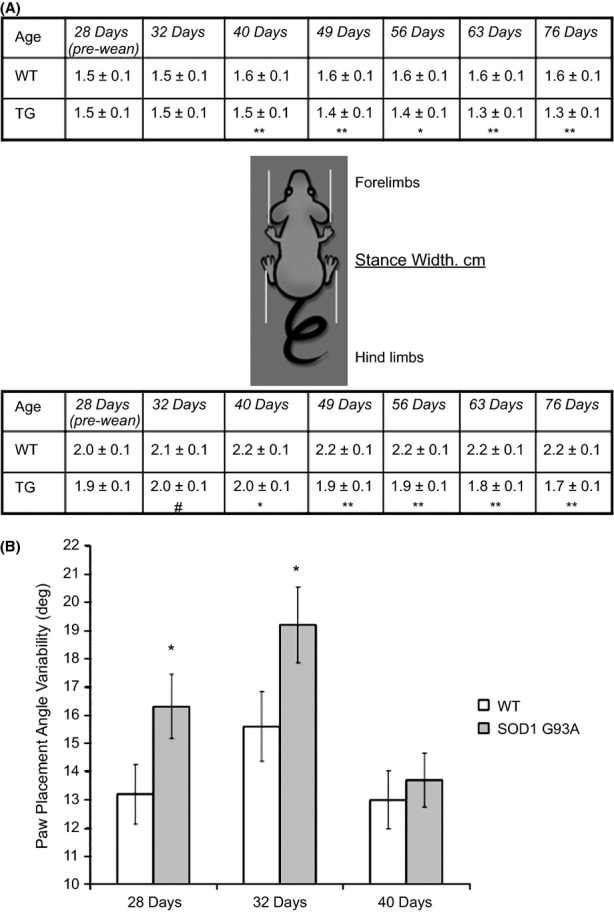
SOD1^G93A^ mice exhibit deficits in motor function that correlate with early muscle denervation. (A) Schematic of forelimb and hindlimb stance width in WT and SOD1^G93A^ mice walking 40 cm/sec up an incline (∼15 degrees). Forelimb stance width is typically more narrow than hindlimb stance width in B6 mice, common to both WT and mutant mice. Narrowing of stance width, a robust and significant phenotype by 7 weeks of age, was evident in SOD1^G93A^ mice by P32. Data were pooled from two cohorts of mice (*n* = 14 per group) and are expressed as mean ± SEM. #*P* < 0.06; *P < 0.05; **P < 0.01. (B) The step-to-step placement of the paws during peak loading of the hindlimbs was significantly more variable in SOD1^G93A^ mice than in WT mice, suggesting aberrant motor function control of the ankles (gastrocnemius and tibialis anterior muscles). *P < 0.05. ANOVA was used to test for statistical differences among WT and SOD1 G93A mice at each age. When the *F*-score exceeded *F*critical for *α* = 0.05, we used post hoc unpaired Student's two-tailed *t*-tests to compare group means. Gait indices between forelimbs and hindlimbs within groups were compared using paired Student's two-tailed *t*-tests.

Previous gait analysis of SOD1^G93A^ mice have indicated supranormal gait prior to neurodegeneration and the onset of gait disturbances at ∼13 weeks of age, when the animals were tested walking horizontally at speeds of 24 and 36 cm/sec (Amende et al. 2005). We did not detect any overt deficits in gait when SOD1 mice voluntarily traversed the walking compartment floor. Initial clinical symptom onset of ALS in patients often occurs as small and subtle changes in muscle strength (e.g., occasional foot drop, difficulty turning a key, slurring of speech). It is difficult to assess these kinds of changes by simple observation of mouse behavior. We therefore challenged the animals with a more rigorous treadmill walking protocol. The treadmill, walking compartment, and camera system were pitched at an angle so that the animals walked up an incline of 15 degrees and the motor speed was set to 40 cm/sec. Under these conditions there was a significant increase in the variability of hindlimb paw placement angle in SOD1 mice at P28 and P30 (Fig. 23). We believe that the variability of hind paw placement angle corresponds to muscle weakness due to initial denervation that occurs in the TA as reported in this study and medial gastrocnemius muscle in a previous study (Gould et al. 2006). These behavior changes may also reflect denervation in other hindlimb muscles that were not studied (e.g., extensor digitorum longus). The difference in paw placement angle tends to disappear by P40. At the same time the differences in hindlimb stance width become more prominent and increases with age. There was also an apparent decrease in hindlimb stance width at P32, although the difference between SOD1 and WT was not statistically significant until P40; a decrease in forelimb stance width was also detected at day 40 (Fig. 23). These more profound changes correspond with increased muscle denervation that occurs with disease progression.

**Figure 24:**
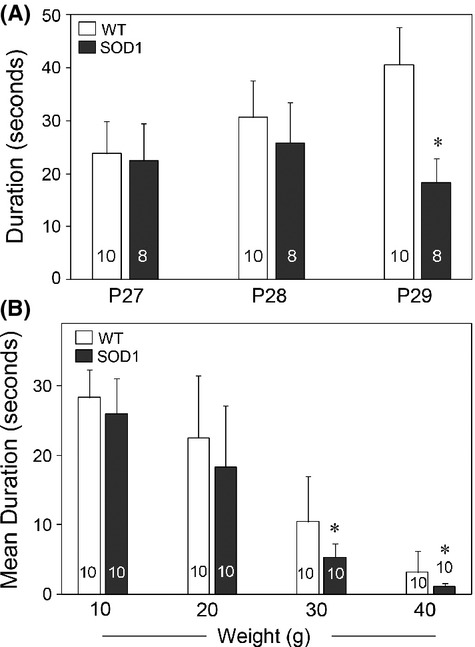
Performance of WT and SOD1 mutant mice on the loaded grid test. Performance is based on the duration of time in seconds (sec) before the loaded grid was dropped. (A) Each mouse was tested twice with a 15 g weight and allowed unlimited time before dropping the weight with a 10-min intertest interval. The values represent the average of two trials/mouse. Results are expressed as mean ± SEM (*n* = 10 animals each phenotype). *P < 0.02, statistical significance was determined using unpaired *t*-tests. (B) Performance of WT and SOD1 mutant mice. Each mouse was tested twice with each weight for a 30-sec fixed duration with 15 sec between each weight and 10 min between the two tests. The values represent the average of the two tests/mouse. Results are expressed as mean ± SEM; the number of animals for each condition is indicated in the bars of the graph. *P < 0.05 as determined using unpaired *T*-test.

Using the loaded grid test as an assay of forelimb muscle strength, SOD1 mutant mice at P29 (but not at P27 or P28) exhibited the first signs of muscle weakness as indicated by a significantly decreased duration of time before dropping a 15 g weight. At P30–31, both WT and SOD1 mice were able to hold a 10 g and 20 g weight for an equivalent duration, but when tested with 30 g and 40 g weights the mutant mice held the weights for a significantly shorter time (Fig. 24).

## Discussion

Sporadic ALS and SOD1 mutant forms of FALS are clinically indistinguishable. Mice and rats expressing mutant forms of human SOD1 develop progressive MN degeneration and clinical signs that closely mimic sporadic and familial forms of human ALS (Gurney et al. 1994). Initial characterization of the animal models understandably focused on pathological events associated with obvious behavioral symptoms (e.g., leg tremor) and MN degeneration.

The onset of overt clinical symptoms in the SDO1^G93A^ mouse is generally reported to occur at approximately P90, but see (Mancuso et al. 2011; Mead et al. 2011; Gerber et al. 2012). We find that signs of axonal degeneration are evident by P75, but the absolute number of ventral root axons is still comparable with WT. The MNs that will die can be identified by P60 even though their removal from the spinal cord does not occur until later ages. Consistent with previous reports, we confirm that muscle denervation long precedes activation of cell death pathways and the classical definition of clinical pathology (Frey et al. 2000; Raff et al. 2002; Medana and Esiri 2003; Fischer et al. 2004; Gould et al. 2006; Palop et al. 2006; Pun et al. 2006; Conforti et al. 2007; Gould and Oppenheim 2007). However, our observations place the onset of denervation at P25–30, 10–20 days earlier than previous reports. As demonstrated previously (Pun et al. 2006; Hegedus et al. 2007), denervation does not occur uniformly in all muscles but is dependent on fiber type with fast fibers being denervated before slow fibers. Here, we report that denervation of the TA muscle occurs between P14 and P30, while at the same time little denervation occurs in the soleus muscle that is composed primarily of slow fibers. Muscle denervation during the first postnatal month calls into question previous characterization of the SOD1^G93A^ mouse model in terms of disease onset. Traditionally, disease onset was considered to occur in the third postnatal month, a time coincident with detection of MN cell death; however, here we also provide evidence that motor function deficits begin coincident with initial muscle denervation. Muscle strength, as assessed by the loaded grid test and treadmill gait was impaired in mutant mice beginning around P30–40 and treadmill deficits only occurred in mice walking uphill at increased speeds when the TA muscle is increasingly engaged (Roy et al. 1991). Symptom onset would be expected to occur when pathological and motor function deficits are evident, and therefore in the SOD1^G93A^ mouse, symptom onset must be considered to occur at P30, rather than at P70–90 as commonly reported. Decreases in muscle strength and the electrophysiological indication of denervation are hallmark signs of clinical symptoms in ALS patients. Furthermore, ALS El Escorial diagnosis criteria include signs of upper and lower MN degeneration, with symptom onset progressing to another area (e.g., lower to upper limbs). Our results provide further evidence that the mutant SOD1^G93A^ mouse reliably models the human disease with pathological signs in both lower and upper MNs (Ozdinler et al. 2011), as well as muscle weakness in both upper (loaded grid test) and lower (gait analysis) limbs. We propose that P30 therefore represents a more realistic approximation of symptom onset in mutant mice and therefore a reevaluation of previous preclinical studies for ALS should be considered in light of this. Indeed, studies where treatment of SOD1 mutant mice was begun at P30 or earlier demonstrated some of the most effective survival promoting effects reported (Kieran et al. 2005; Pun et al. 2006; Gifondorwa et al. 2007).

### Muscle denervation as a therapeutic target

Several studies have demonstrated that protecting cell bodies from death fails to substantially alter disease progression or life span (Sagot et al. 1995; Kostic et al. 1997; Ferri et al. 2003; Chiesa et al. 2005; Libby et al. 2005; Gould et al. 2006; Suzuki et al. 2007). Rather, the early loss of connectivity may be the most important contribution to the organisms disability and this aspect of neurodegenerative disease has been a neglected potential therapeutic target.

### What leads to denervation?

There is no difference at P14 between SOD1 and WT TA muscle in terms of the number of innervated postsynaptic NMJs. This result suggests that NMJs are initially formed normally, but by P30 there is a significant denervation of TA NMJs. Our results are consistent with previous reports in the literature indicating that FF MNs (e.g., TA MNs) are more susceptible to denervation than S MNs (e.g., soleus MNs) (Frey et al. 2000; Hegedus et al. 2007; Pun et al. 2006). The early denervation of FF MNs is partially compensated for functionally by sprouting and reinnervation by fatigue resistant (FR) and slow (S) MNs (Frey et al. 2000; Hegedus et al. 2007). Although it has been suggested that eventually even these more resistant MN subtypes become unable to compensate at which point muscle weakness ensues followed by MN degeneration (Hegedus et al. 2007), there appears to be a fast fiber-specific vulnerability in the SOD1 mouse. Accordingly, it is important to understand the mechanisms involved in the differential vulnerability of MNs innervating fast fatigable (FF), FR, and S muscle fibers in ALS. We find that the presynaptic terminals and axons of both TA and soleus innervating MNs show enlarged and vacuolated mitochondria. Although we did not observe a loss of presynaptic vesicles in the SOD1 mice at P30, presynaptic vesicles were reported to be completely lost early in disease (P40–42; Pun et al. 2006), this result may reflect a loss of antigenicity rather than an actual physical loss of vesicles. Because many of the pathological events observed in the mutant spinal cord appear to occur in both TA and soleus motor pools, there must be some specific trigger that preferentially causes FF MNs to be more susceptible to early pathogenesis. The physiology and connectivity of FF MNs are possible candidates involved in this increased vulnerability (Saxena et al. 2009).

One proposed hypothesis is that vulnerable neurons in some way fail to compensate for disease-related conditions. However, there are no reports of differential expression of mutant SOD1 in vulnerable motor pools. Although MNs do not appear to mount the typical stress response in terms of increasing expression of Hsp70 (reviewed in Robinson et al. 2011), it is not known whether MNs from different motor pools respond differently in other ways to stressful stimuli (e.g., Saxena et al. 2009).

### Axonal transport and muscle denervation

Deficits in axonal transport have been reported in the ALS mouse model and likely contribute to MN dysfunction and pathology (Williamson and Cleveland 1999; Rao and Nixon 2003; Jablonka et al. 2004). Indeed, mutant SOD1 can disrupt the cytoplasmic dynein motor in MNs (Ligon et al. 2005), suggesting a direct mechanism by which mutant SOD1 can alter retrograde transport and synaptic stability. Our results suggest that changes in retrograde transport do not occur prior to early denervation in the TA muscle (also see Bilsland et al. 2010; Marinkovic et al. 2012). Therefore, deficits in retrograde transport alone do not appear sufficient to mediate muscle denervation and MN dysfunction. Further evidence for this comes from experiments using the Loa (Legs at odd angle) mice that have a mutation in cytoplasmic dynein. This mutation results in deficits in retrograde transport, and mild neuronal degeneration (Hafezparast et al. 2003). Surprisingly, when the Loa and SOD1^G93A^ mice are crossed, there is an amelioration of disease and enhanced survival (Kieran et al. 2005); although when the Loa mice are crossed with the SOD1^G85R^ or SOD1^G37R^ mice there is no change in survival (Ilieva et al. 2008). These results in the SOD1^G93A^ model suggest that inhibition of retrograde transport may be protective, possibly by inhibiting the transport of toxic negative signals. During development it is well established that MN survival is dependent on target-derived trophic support (Oppenheim et al. 2013); however, our recent report suggests that muscle can also regulate MN survival by the production of prodeath factors such as pro-brain dervived neurotrophic factor (Taylor et al. 2012). Additionally, there appears to be a fiber type-specific retrograde influence on MN innervation (Chakkalakal et al. 2012). During development all MNs will express the synaptic vesicle protein SV2A; however, those MNs that innervate fast fibers downregulate SV2A expression and only MNs innervating slow fibers maintain SV2A expression. The authors also showed that when fast fibers are converted to a slow phenotype, the MNs innervating those fibers express SV2A indicating a retrograde fiber type-specific signal that induces MN phenotype.

In the SOD1 mouse slow muscle fibers may produce more MN survival promoting factors as compared with fast fibers. In support of this theory, slow fibers have been shown to express higher levels of Hsp70 as compared with more vulnerable fast fibers (Locke et al. 1991, 1994; Gifondorwa et al. 2012), and administration of recombinant Hsp70 can maintain muscle innervation, delay symptom onset, and extend survival of SOD1^G93A^ mice (Gifondorwa et al. 2007). Alternatively, slow muscles that contain more mitochondria may be better able to compensate for the increased oxidative stress shown to occur in the mutant SOD1 mice (e.g., Jang et al. 2010). Fast muscles may produce negative factors, including enhanced oxidative stress that promote NMJ dysfunction and denervation (e.g., Perlson et al. 2009).

### Empty cytoplasmic vacuoles

The accumulations of small, empty vacuoles in mutant MN cytoplasm are observed by day 14. The vacuoles become more numerous by day 30 and at later stages the cytoplasm is full of these vacuoles. We are unable to definitely determine the source of these vacuoles; however, it is unlikely that the vacuoles are an artifact of fixation as they were unique to SOD1^G93A^ animals and not observed in WT animals.

Similar vacuoles have been reported to originate from ER and may result from an unfolded protein response or ER stress (Ilieva et al. 2007; Nagata et al. 2007; Nishitoh et al. 2008; Kanekura et al. 2009). For example, vacuolization of the rER has been shown to occur in MNs following chronic excitotoxicity (Tarabal et al. 2005). Indeed, MNs that appear to be most susceptible in ALS, those that innervate fast, fatiguable muscle fibers, have been shown to initiate an unfolded protein response at early as day 25 (Saxena et al. 2009), corresponding to the time when we begin to observe increased numbers of vacuoles. However, we did not observe vacuoles or other morphological changes selectively in MNs that innervate fast fibers, and we never observed structural perturbations of rER, even at late stages, although it is possible that the vacuoles originate from the smooth ER.

We also observed small, empty vacuoles near the Golgi apparatus, suggesting either *cis*- or *trans*-Golgi elements as a possible source. These findings may indicate an early breakdown of cisternal maturation of Golgi membranes as previously suggested to occur in ALS mouse models (Gonatas et al. 1992, 2006; Mourelatos et al. 1996; Stieber et al. 1998; Martinez-Menárguez et al. 2001; Schaefer et al. 2007; Fan et al. 2008). Interestingly, small cytoplasmic vacuoles similar to those we observed were also found in MNs of mice with a type C RNA viral infection (Gardner et al. 1973; Andrews and Gardner 1974). Because these vacuoles did not possess viral particles, they may indicate a cellular stress response to the infection.

We also observed the small vacuoles adjacent to mitochondria suggesting another possible source, namely from budding of the outer mitochondrial membrane. Mutant SOD1^G93A^ as well as ubiquitin have been shown to accumulate in mitochondria, possibly impairing function (Jaarsma et al. 2001; Sasaki et al. 2004). Furthermore, accumulation and aggregation of SOD1^G93A^ has been suggested to cause mitochondrial vacuolization through expansion of the intermembrane space (Higgins et al. 2003). However, the small cytoplasmic vacuoles located adjacent to mitochondria often appeared as distinct profiles rather than as “buds” from the mitochondria.

### Mitochondria

Mitochondrial abnormalities and/or dysfunctions have been proposed to play key roles in the pathology of ALS (see Manfredi and Xu 2005; Magrané and Manfredi 2009; Shi et al. 2010; Martin 2010; Schon and Przedborski 2011 for reviews). Alterations in nutrient availability, increases in oxidative stress, unfolded protein responses, mutant proteins, and other cellular stresses place increased demand on and possible damage to mitochondria. Furthermore, mitochondrial DNA repair enzymes are reduced in the spinal cord of mutant SOD1 mice (Murakami et al. 2007). Swollen and vacuolated mitochondria and mega-mitochondria were the most notable features we observed in the spinal cord of postnatal mutant mice and were first observed at P7. While these abnormalities were found in the presynaptic terminals of NMJs, in MN soma, and in presynaptic terminals of axo-somatic synapses, they were most abundant in MN dendrites. This observation confirms and extends an earlier report of such abnormalities being present already at P12 in this mouse model with the most severe changes observed in MN dendrites (Bendotti et al. 2001). Liver mitochondria subjected to environmental stresses initially respond with an apparent increase in fusion to become mega-mitochondria (Wakabayashi 2002). If the stress is limited, the mitochondria are reduced to prestress size whereas, if the stress is maintained, the mitochondria go on to become greatly enlarged and vacuolated. We propose that the presence of mega-mitochondria on P7 in our material may be an adaptive response to pathology, with failure to correct the insult leading to further mitochondrial swelling and cytochrome C release.

Alterations in mitochondria have been proposed to initiate symptom onset in the mutant SOD1^G93A^ mice (Kong and Xu 1998), although that study used a definition of symptom onset that occurs much later than the earliest time point observed in the current study. Mitochondrial fission and fusion are ongoing, normal events, with mitochondrial fission playing a critical role in synapse formation in cultured hippocampal neurons (Li et al. 2008). Both fission and fusion are increased by cellular stress. Although aberrant fusion of mitochondria is thought to contribute to the formation of mega-mitochondria, defects in fission may also play a role. Considering that our results indicate a decrease in the total number of mitochondria, but a substantial increase in their size in MNs of SOD1^G93A^ mice, increased fusion and/or decreased fission may contribute to some of the earliest signs of pathology. The proapoptotic gene Bax may be a critical mediator of this process. In Bax knockout/SOD1^G93A^ mice, the appearance of enlarged, vacuolated mitochondria is significantly delayed as is initial muscle denervation and subsequent stages of pathogenesis (Gould et al. 2006). Survival was extended modestly in these animals indicating that while mitochondrial dysfunction related to enhanced mitochondrial fusion may be related to early denervation, it is not the only mediator of disease. Furthermore, many of the observed changes in mitochondria of our mutant mice are also observed in hSOD1WT transgenic mice (although not to the same extent), but these mice do not express the same pathogenesis as the SOD1^G93A^ mice (Jaarsma et al. 2000).

### Synapses

We observed a significant decrease in axo-somatic type I “excitatory” synapses on mutant MNs and an increase in C-terminals, whereas there was no change in the number of type II “inhibitory” synapses or in the number of total synapses in P30 SOD1 ventral spinal cords. Axo-dendritic type I “excitatory” synapses in the white matter were reduced in mutant mice. The decrease in type I synapses is reflected in the decrease in the total number of axo-dendritic synapses. Interestingly, in the SMA mouse model, a decrease in excitatory input is also observed on dendrites and soma, while there was no apparent change in inhibitory input (Lin and Koleske 2010; Mentis et al. 2011). In the SMA mouse at earlier postnatal ages, there was no difference in synapse number between the SMA versus control mice, suggesting that the spinal cord circuitry is capable of forming new synapses, but not maintaining them as disease progresses (Lin and Koleske 2010). In terms of white matter synapses, our results at P14 are somewhat different in that we detected an increase in the total number of synapses on white matter dendrites in SOD1 animals versus WT. This increase in axo-dendritic synapses at P14 is consistent with the increased number of axons observed at this age. We propose that the differences in synapse and axon number observed at P14 may indicate an alteration in axonal pruning that occurs in early development and that the increase in the number of glial cells at this age may reflect a delay in axonal pruning and/or myelination.

Excitatory cholinergic C-terminals are present on MN soma and proximal dendrites and were identified 40 years ago (Conradi and Skoglund 1969; Nagy et al. 1993; Li et al. 1995). Their origin and function was not known until recently when they were determined to originate from a small group of cholinergic interneurons located near the central canal (Frank 2009; Zagoraiou et al. 2009). These terminals appear to increase MN excitability via mAChRs (M2), potentiating the strength of muscle contraction. Our results confirm a previous report that found an increase in C-terminal coverage on MNs in SOD1 mice (Pullen and Athanasiou 2009). These investigators suggest the increase in C-terminal coverage reflects a mechanism by cholinergic interneurons to compensate for the loss of excitatory input. Indeed, changes in lumbar MN excitability have been reported to occur as early as the second postnatal week in SOD1^G93A^ low expressor and SOD1^G85R^ mutant mice (Pambo-Pambo et al. 2009). Furthermore, the earliest pathological change reported in SOD1^G93A^ mice is increased hyperexcitability in neonatal MNs (van Zundert et al. 2008). Together these results may indicate altered synaptic activity as an early event mediating pathology. Interestingly, MNs innervating fast-twitch muscles are reported to have almost twice the number of C-terminals compared with MNs that innervate slow-twitch muscles that are less vulnerable to SOD1 pathogenesis, and ocular muscles whose MNs are spared in ALS have no C-terminal synapses.

Changes in synaptic function are involved in neuronal plasticity that allows neurons to adapt to alterations in their environment in both health and disease. Functional synapses are critical not only for neuronal survival, but also for survival of the organism. For example, mutations that affect synaptic vesicle trafficking (such as SV2 or synaptic vesicle proteins such as CSP-α) result in neonatal or early postnatal lethality (see Gould and Oppenheim 2007 for review). Many abnormal proteins that are associated with neurodegenerative diseases interfere with function and integrity of pre- and/or postsynaptic components of synapses by mechanisms that may involve excitotoxicity and oxidative stress (see Palop et al. 2006 and Wishart et al. 2006 for reviews). For these reasons, it is not surprising that alterations of synapses is often observed in psychiatric and neurodegenerative disorders (reviewed in Palop et al. 2006; Lin and Koleske 2010). Interestingly, in the present study the most profound morphological changes in mitochondria were observed on distal dendrites at all ages, and by P30 there was an apparent decrease in the number of total synapses and a significant reduction in type I synapses. Synaptic activity regulates mitochondrial localization, and mitochondria are enriched at pre- and postsynaptic terminals (MacAskill et al. 2010). Furthermore, activation of glutamate receptors is key to halting mitochondria mobility (reviewed in MacAskill et al. 2010). Mature MNs are susceptible to glutamate excitotoxicity (Rothstein et al. 1991; Rothstein 1995; Brunet et al. 2009) that results in excessive increased intracellular calcium at the postsynaptic terminal, causing Ca^2+^ overload and increased Ca^2+^ uptake by local mitochondria (reviewed in MacAskill et al. 2010). The increased Ca^2+^ from activation of glutamate receptors also results in increased nitric oxide production (Rintoul and Reynolds 2009). As discussed above, these events place increased demand on mitochondria that may then form mega-mitochondria as a compensatory mechanism. Mutant SOD1 protein appears to interfere with normal fission and fusion events, further compromising mitochondrial function. These events appear to be perpetuated, eventually leading to the greatly enlarged and presumably dysfunctional mitochondria.

This proposed series of events is consistent with the glutamate toxicity hypothesis of ALS. By contrast, our results of decreased numbers of type I “excitatory” synapses appears difficult to reconcile with this hypothesis. However, we only examined synapses at P30, a time when swollen and vacuolated mitochondria were routinely found in distal and proximal dendrites. Mutant SOD1 is thought to alter the development of electrical properties of MNs resulting in hyperexcitability at early postnatal ages (Amendola et al. 2007; Pambo-Pambo et al. 2009). It is quite possible that mitochondrial dysfunction due to the mutant SOD1 protein, together with other environmental stressors, initially occurs as early as P7, so that even normal levels of glutaminergic synapses may result in hyperexcitability due to the increased intracellular Ca^2+^, further increasing functional demands on mitochondria. Loss or dysfunction of mitochondria in postsynaptic sites has been shown to result in decreases in morphological plasticity and dendritic spine formation as well as eventual loss of spines and synapses (reviewed in MacAskill et al. 2010). Therefore, excitotoxicity may begin as early as the first postnatal week, one consequence of which is a subsequent decrease in excitatory synapses by day 30.

### Glia

Astrocytes and microglia exhibit a profound response in motor areas of both patient and mouse models of ALS (for examples see, McGeer et al. 1993; Schiffer et al. 1996; Hall et al. 1998). Results suggesting that ALS is a cell nonautonomous disorder have reinforced the idea that glial cells are either affected by or contribute to disease pathology (Barbeito et al. 2004; Pehar et al. 2005; Sargsyan et al. 2005; Boillée et al. 2006; Monk and Shaw 2006; Jullien 2007; Henkel et al. 2009; Ilieva et al. 2009; King et al. 2011). Several studies have suggested that astrocytes directly contribute to MN degeneration possibly through altered function or secretion of specific factors (Pehar et al. 2004; Domeniconi et al. 2007; Nagai et al. 2007). Astrocytes have also been shown to undergo apoptosis in the SOD1^G93A^ mouse model (Rossi et al. 2008). Both cytotoxic (M1) and neuroprotective (M2) microglia contribute to disease progression, and the mutant SOD1 protein has been shown to promote a transition from M2 to M1 microglia in mouse models (see Henkel et al. 2009 for review). Interestingly, at no time point examined by us, including end stage, were the ultrastructural pathological abnormalities seen in MNs observed in glial cell bodies, although at later stages, astrocytes expressed increased filaments, a normal indication of activation. We did not observe glial cell mitosis or degeneration, although at the ultrastructual level these events may have been missed. While we did not detect degeneration of oligodendrocytes, disruption of myelin was frequently observed and the number of oligodendrocytes appeared to increase steadily in mutant mice. This result is in agreement with a recent report that NG2 cells retain commitment to oligodendrocytes lineage in normal CNS as well as in the spinal cords of ALS mice (Kang et al. 2010), although the specific signals that promote increased oligodendrocytes are not known. Our results suggest that while glial cells react to pathological alterations in MNs, the response of glial cells does not appear to include the same pathological morphological changes observed in MNs.

## Summary

The ultrastructural morphology that we observed in MN soma and dendrites is not consistent with that reported following axotomy, polioviral infection, strychnine or cobra venom administration, or mercury poisoning (Bodian 1964; Chang and Hartmann 1972; Yates and Yates 1972; Johnston and Sears 1989). The presence of mega-mitochondria as well as swollen and vacuolated mitochondria is also observed in MNs of asphyxiated spinal cord of cat and in superior mesenteric-celiac ganglia of aged and diabetic mice (Van Harreveld and Khattab 1967; Schmidt et al. 2008). Taken together, our results suggest that the MN response to injury versus ALS pathology is not the same and caution should be used when comparing the two. Additionally, the presence of enlarged mitochondria is in agreement with other pathologies that involve metabolic stress, suggesting that in the ALS mouse model initial pathology is in response to a metabolic stress that may result from multiple stimuli (Saxena et al. 2009).

Although it is tempting to speculate that a single insult can precipitate disease pathology, our current examination of ultrastructural pathological changes failed to identify such an initiating event. It is clear, however, that alterations in mitochondria morphology and presumably their function are one of the earliest pathological events we observe, perhaps in response to an even earlier imbalance of synaptic input on MNs, occurring long before and therefore not likely to be a proximate causal factor in precipitating functional or physical loss of MNs. More likely, these events reflect a response of the MN to potentially toxic changes in intracellular or extracellular environments that gradually results in muscle denervation, muscle weakness, and eventual loss of MNs, paralysis, and death.

Taken together, our study together with previous reports characterizing disease pathogenesis in mutant SOD1 fALS mice have revised the traditional view of ALS as a disease of the cell body. However, despite our extensive characterization of early events in the spinal cord and at the NMJ in the SOD1^G93A^ mouse, we still cannot conclusively state that disease begins at one site versus the other. We can conclude, however, that while the disease process is not cell autonomous, MNs are a key site of initial pathology (disease onset). Furthermore, we show that there is significant pathology, including muscle denervation and weakness during the first postnatal month, suggesting that the onset of disease occurs significantly earlier than previously reported.

**Figure 25:**
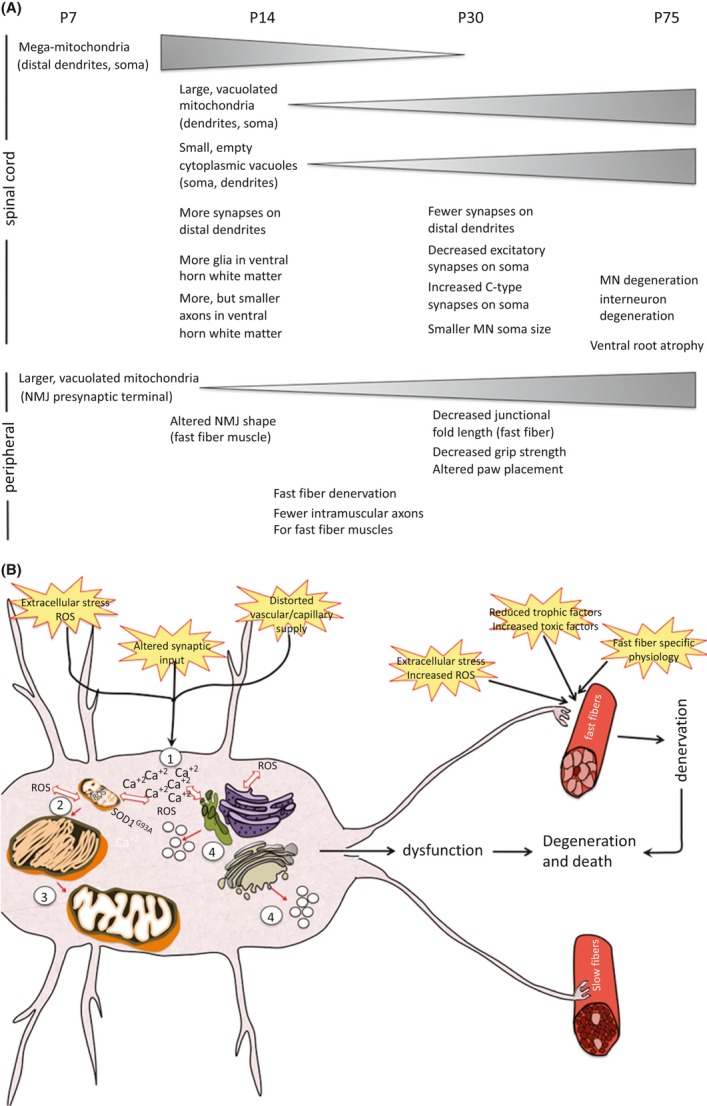
(A) A summary of pathological events in central and peripheral components of the neuromuscular system of SOD1^G93A^ mice and the time of their appearance is shown (see accompanying paper (doi: 10.1002/brb3.143) for description of pathology in the spinal cord). Triangles show either increases or decreases in the pathology over time. (B) A proposed sequence of initial pathology in MNs in SOD1^G93A^ mice is shown. Altered synaptic input either alone or with a perturbed capillary supply and extracellular stress results in an imbalance of Ca^2+^ within the MN (1). This imbalance results in intracellular generation of reactive oxygen species (ROS) and together with the presence of the mutant SOD1 protein creates an environment that results in the formation of mega-mitochondria (2). The mitochondria are not able to resolve the “stress,” possibly because of their impairment by mutant SOD1, and thus become vacuolated (3). The imbalance of intracellular Ca^2+^ and generation of ROS can also initiate the unfolded protein response and ER stress resulting in vacuolization of smooth ER and Golgi (4). Together, these events result in MN dysfunction. MNs innervating fast muscle fibers also encounter events at the NMJ including extracellular stress and ROS, fast fiber-specific physiology and possibly a reduced supply of trophic factors or increased toxic factors that lead to muscle denervation. The combination of the denervation and MN dysfunction eventually leads to MN degeneration and death. While not shown in the diagram, the events contributing to MN dysfunction in the spinal cord most likely contribute to glial cell activation and/or dysfunction (e.g., decrease in astrocyte glutamate transporter) that further enhances disease pathology. With the exception of the vascular system, also not shown here are noncell (MN) autonomous contributions that may contribute to disease onset (e.g., astrocytes, Schwann cells).

We have developed a proposed sequence of events that mediate disease pathology in the SOD1^G93A^ mouse based on the results presented here (Fig. 25). However, although there is now a growing consensus in the field that the axon and synapses are the first cellular sites of degeneration, it is not known whether NMJ denervation is initiated autonomously at that site or by pathology in the cell body, in nonneuronal cells or even in non-MNs (Bettini et al. 2007; Conforti et al. 2007; Gould and Oppenheim 2007; Zhong et al. 2008, 2009; Yoshikawa et al. 2009). The specific molecular mechanisms mediating axon/synapse loss in ALS are still largely unknown (Saxena and Caroni 2007). Nonetheless, our results together with previous studies from other laboratories suggest new avenues for investigations that may provide novel targets for therapeutic interventions.
